# Supplementary material for: Aptamer-assisted tumor localization of bacteria for enhanced biotherapy
Source: Nat Commun. 2021 Nov 15;12:6584. doi: 10.1038/s41467-021-26956-8 (PMC8593157; doi:10.1038/s41467-021-26956-8)
Supplement: Supplementary file 1 — Supplementary Information [file 41467_2021_26956_MOESM1_ESM.pdf]

Supplementary Information for

Aptamer-assisted tumor localization of bacteria for enhanced biotherapy

Zhongmin Geng[1], Zhenping Cao[1], Rui Liu[1], Ke Liu[1], Jinyao Liu[1,2], Weihong Tan[1]

[1]Shanghai Key Laboratory for Nucleic Acid Chemistry and Nanomedicine, Institute of Molecular Medicine, State Key Laboratory of Oncogenes and Related Genes, Renji Hospital, School of Medicine, Shanghai Jiao Tong University, Shanghai 200127, China.

[2]Shanghai Key Laboratory of Gynecologic Oncology, Renji Hospital, School of Medicine, Shanghai Jiao Tong University, Shanghai 200127, China.

Correspondence and requests for materials should be addressed to J.L. (email: jyliu@sjtu.edu.cn)

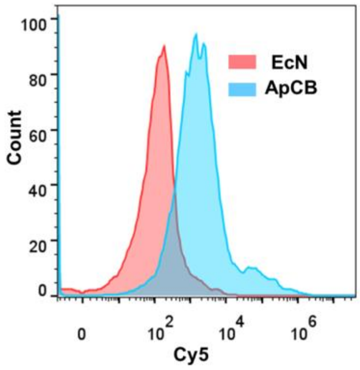

Supplementary Figure 1. Flow cytometric analysis of EcN and ApCB by tracking the fluorescent intensity of Cy5-labelled AS1411. Source data are provided as a Source Data file. Flow plots are representative of 5 independent biological samples.

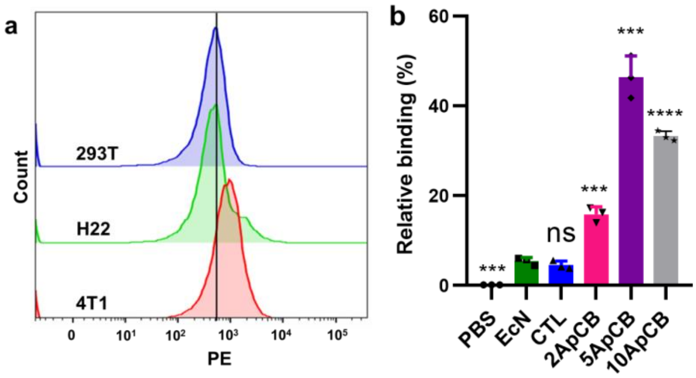

Supplementary Figure 2. (a) Flow cytometric analysis of surface nucleolin on 293T, H22, and 4T1 cells after incubation with PE-conjugated anti-nucleolin antibodies. Flow plots are representative of 3 independent biological samples. (b) Percentages of 4T1 cells binding with $ApCB_{GFP}$ after co-incubation at 37 °C for 2 hours. Error bars represent the standard deviation (n = 3 independent experiments). Data are presented as mean values ± SD. Significance was assessed using Student's t-test (two-tailed), giving p-values: 0.000004 for 10ApCB *vs* EcN; 0.0001 for 5ApCB *vs* EcN; 0.0007 for 2ApCB *vs* EcN; 0.0004 for PBS *vs* EcN. ns: no significance. ***$p < 0.001$, ****$p < 0.0001$. Source data are provided as a Source Data file.

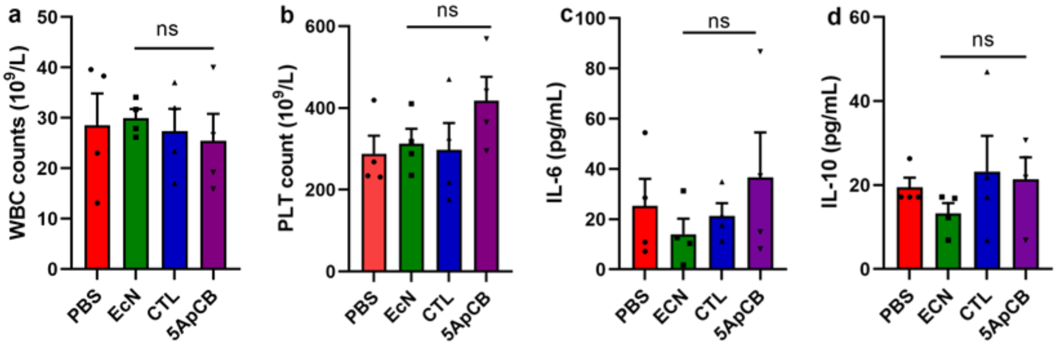

Supplementary Figure 3. Assessment of in vivo inflammatory responses. Routine blood analysis including (a) WBC and (b) PLT counts. Levels of cytokines including (c) IL-6 and (d) IL-10 in serum measured by commercially available enzyme-linked immunosorbent assay (ELISA) kits. Samples were injected through tail vein and blood was withdrawn intraorbitally at 60 hours post-injection. Error bars represent the standard deviation (n = 4). Data are presented as mean values ± SD. Significance was assessed using Student's t-test (two-tailed). ns: no significance. Source data are provided as a Source Data file.

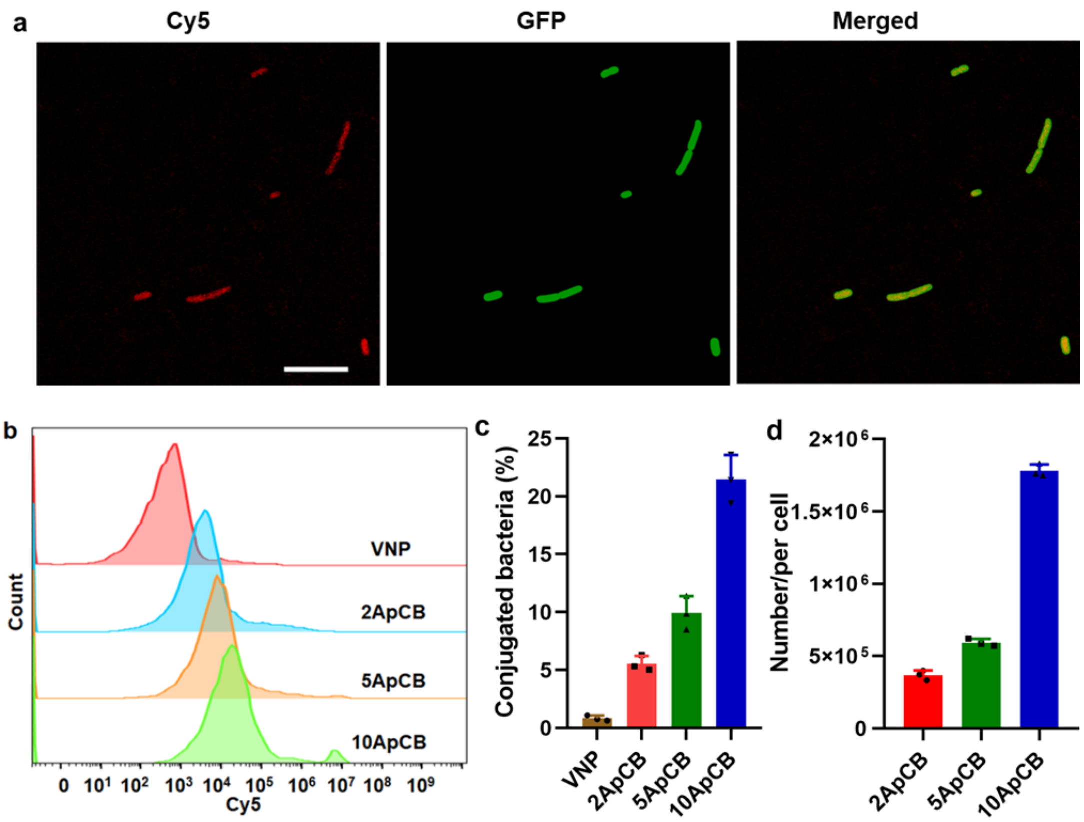

Supplementary Figure 4. Characterization of aptamer-conjugated VNP. (a) Typical LSCM images of aptamer-conjugated VNP. Red and green channels indicate aptamer conjugated with Cy5 and VNP producing GFP, respectively. Images are representative of 3 independent biological samples. Scale bar: 10 μm. (b) Flow cytometric analysis of VNP and VNP conjugated with Cy5-labelled AS1411. Flow plots are representative of 3 independent biological samples. (c) Percentages of conjugated VNP under different feed ratios. Error bars represent the standard deviation (n = 3 independent experiments). Data are presented as mean values ± SD. (d) Average binding number of aptamers on each bacterial quantified by calculating the difference of fluorescent intensity of the aptamer solution after reaction. Error bars represent the standard deviation (n = 3 independent experiments). Data are presented as mean values ± SD. Source data are provided as a Source Data file.

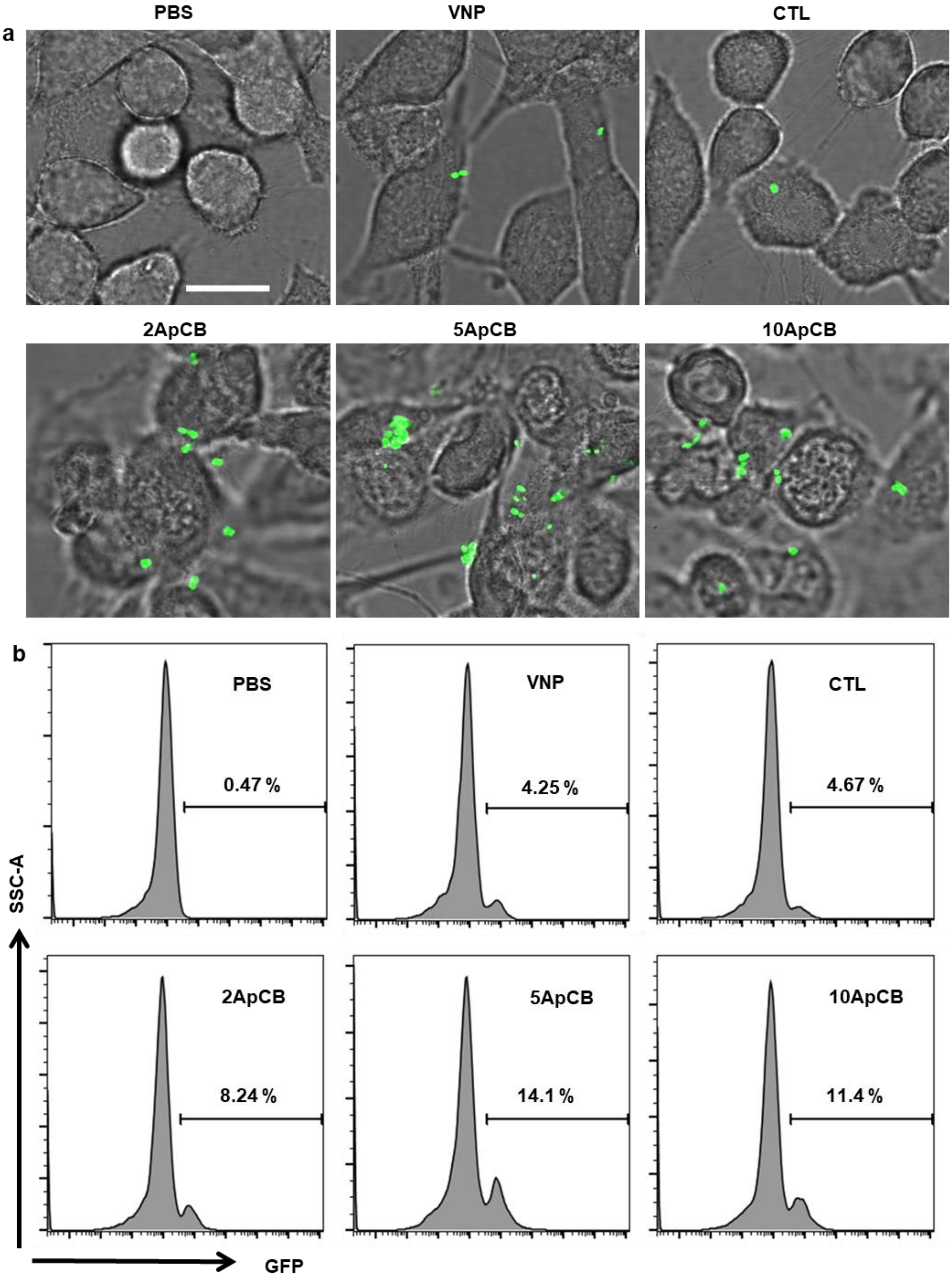

Supplementary Figure 5. Binding of ApCB with 4T1 cells. (a) Representative LSCM images of 4T1 cells after incubation with PBS, VNP_{GFP}, CTL_{GFP}, $2ApCB_{GFP}$, $5ApCB_{GFP}$, and $10ApCB_{GFP}$ at 37 °C for 2 hours, respectively. Cells were rinsed with PBS before observation. Green channel means VNP producing GFP. Images are representative of 3 independent biological samples. Scale bar: 15 µm. (b) Flow cytometric analysis of the co-incubated 4T1 cells. Flow plots are representative of 3 independent biological samples. Source data are provided as a Source Data file.

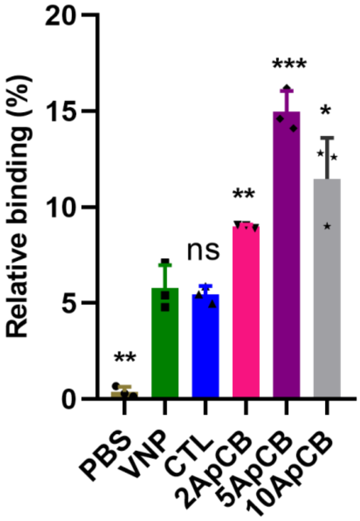

Supplementary Figure 6. Percentages of 4T1 cells binding with $ApCB_{GFP}$ after co-incubation at 37 °C for 2 hours. Cells were quantified by flow cytometric analysis. Error bars represent the standard deviation (*n* = 3 independent experiments). Data are presented as mean values ± SD. Significance was assessed using Student's *t*-test (two-tailed), giving *p*-values: 0.0158 for 10ApCB *vs* VNP; 0.0006 for 5ApCB *vs* VNP; 0.0096 for 2ApCB *vs* VNP; 0.0016 for PBS *vs* VNP. *$p < 0.05$, **$p < 0.01$, ***$p < 0.001$, ns: no significance. Source data are provided as a Source Data file.

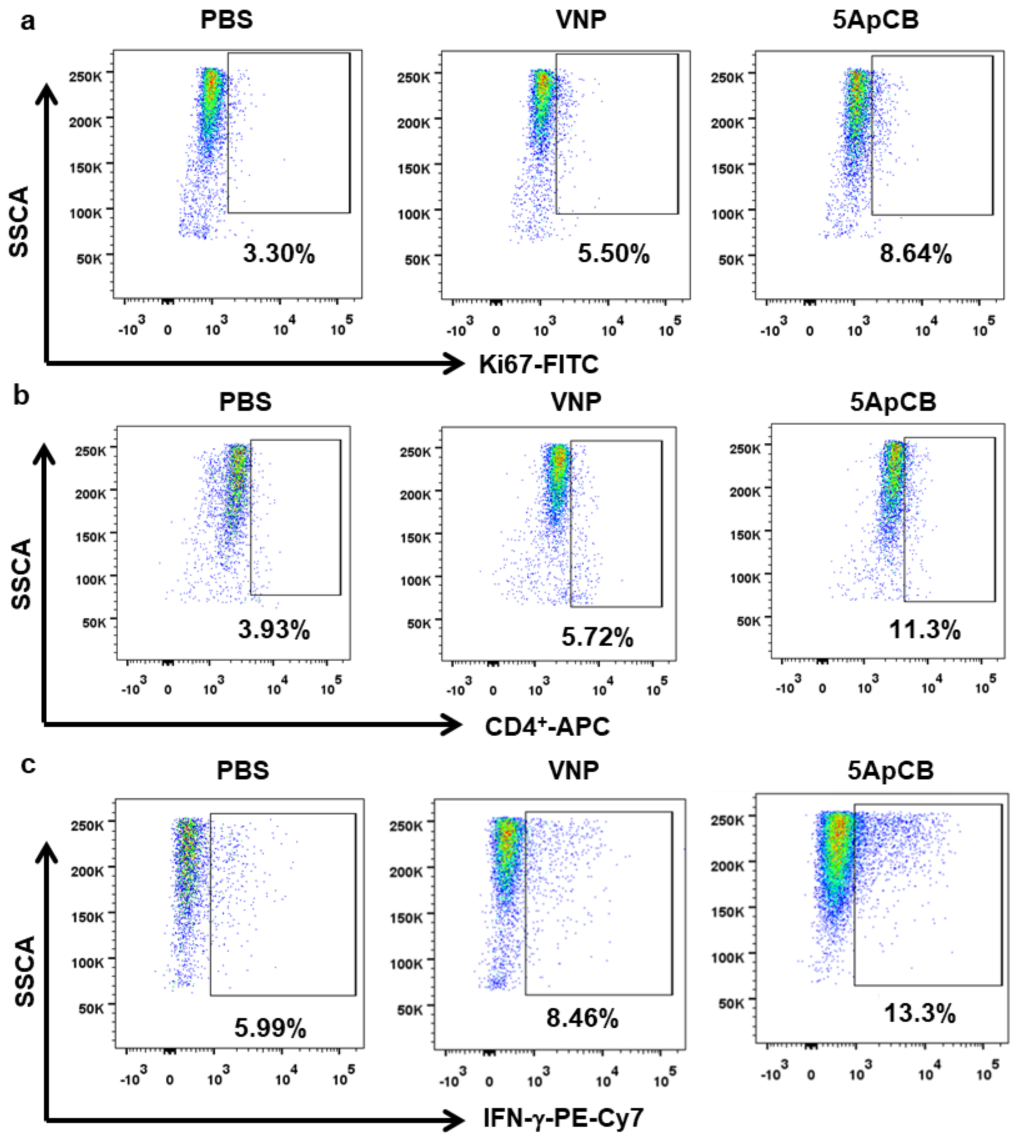

Supplementary Figure 7. (a) Percentage of Ki67, (b) the population of $CD4^+$ T cells, and (c) Percentage of IFN-γ inside the tumors after different treatment. All cells were gated on $CD3^+$ cells. Source data are provided as a Source Data file.

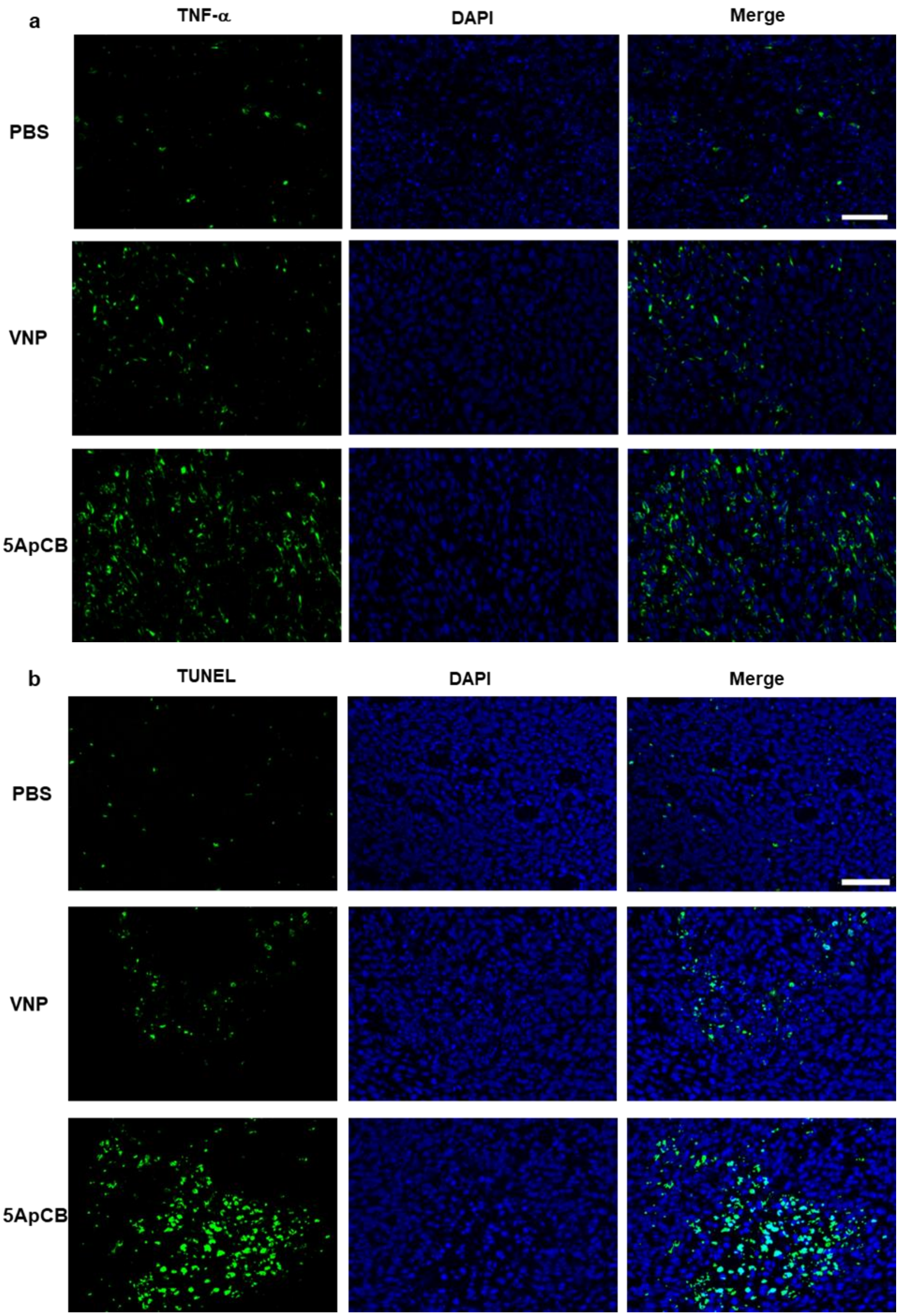

Supplementary Figure 8. Images of tumor tissues stained with (a) TNF-α and (b) TUNEL after different treatments. Images are representative of 3 independent biological samples. Scale bar: 50 μm. Source data are provided as a Source Data file.

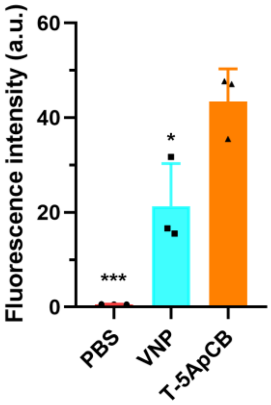

Supplementary Figure 9. Geometric mean fluorescence intensity of H22 cells after co-incubation with T-5ApCB$_{GFP}$. Both PBS and VNP were used as controls. Error bars represent the standard deviation (*n* = 3 independent experiments). Data are presented as mean values ± SD. Significance was assessed using Student's *t*-test (two-tailed), giving *p* values: 0.0004 for T-5ApCB *vs* PBS; 0.0279 for T-5ApCB *vs* VNP. **p* < 0.05, ****p* < 0.001. Source data are provided as a Source Data file.

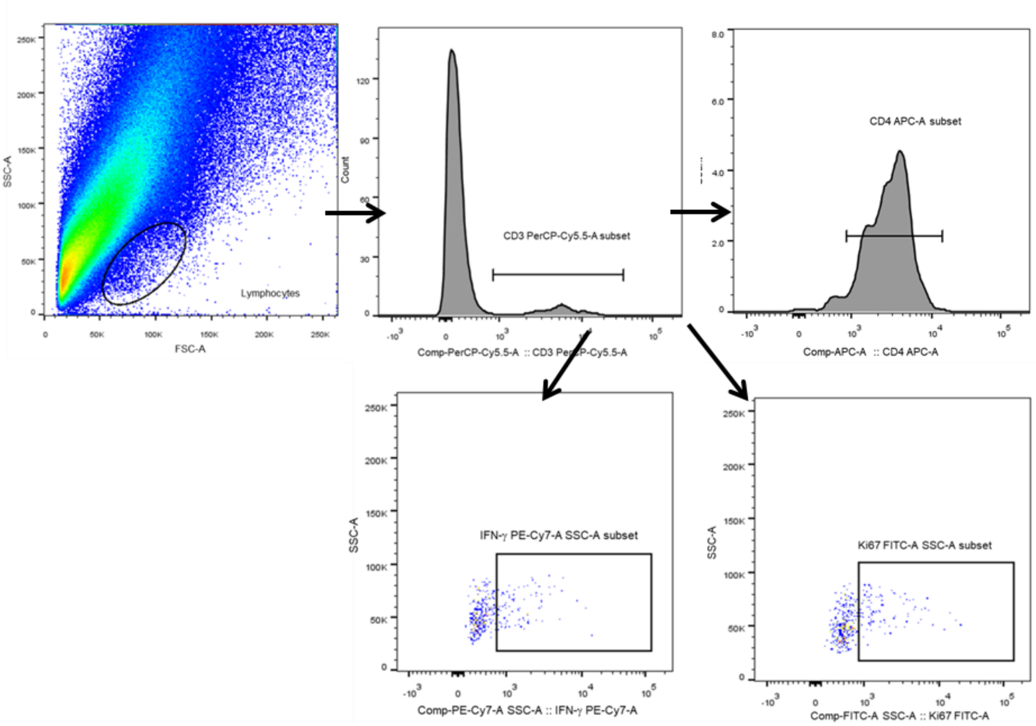

Supplementary Figure 10. Gating strategy to sort CD3$^+$, CD4$^+$, IFN-γ, and Ki67 cells from in vivo tumor cells. Source data are provided as a Source Data file.
