# Supplementary material for: Aptamer-assisted tumor localization of bacteria for enhanced biotherapy
Source: Nat Commun. 2021 Nov 15;12:6584. doi: 10.1038/s41467-021-26956-8 (PMC8593157; doi:10.1038/s41467-021-26956-8)
Supplement: Supplementary file 3 — Reporting Summary [file 41467_2021_26956_MOESM3_ESM.pdf]

nature research

Corresponding author(s): Jinyao Liu

Reporting Summary

Nature Research wishes to improve the reproducibility of the work that we publish. This form provides structure for consistency and transparency in reporting. For further information on Nature Research policies, see Authors & Referees and the Editorial Policy Checklist.

Statistical parameters

When statistical analyses are reported, confirm that the following items are present in the relevant location (e.g. figure legend, table legend, main text, or Methods section).

n/a	Confirmed	
☐	☒	The exact sample size (n) for each experimental group/condition, given as a discrete number and unit of measurement
☐	☒	An indication of whether measurements were taken from distinct samples or whether the same sample was measured repeatedly
☐	☒	The statistical test(s) used AND whether they are one- or two-sided<br>*Only common tests should be described solely by name; describe more complex techniques in the Methods section.*
☐	☒	A description of all covariates tested
☐	☒	A description of any assumptions or corrections, such as tests of normality and adjustment for multiple comparisons
☐	☒	A full description of the statistics including central tendency (e.g. means) or other basic estimates (e.g. regression coefficient) AND variation (e.g. standard deviation) or associated estimates of uncertainty (e.g. confidence intervals)
☐	☒	For null hypothesis testing, the test statistic (e.g. F, t, r) with confidence intervals, effect sizes, degrees of freedom and P value noted<br>*Give P values as exact values whenever suitable.*
☒	☐	For Bayesian analysis, information on the choice of priors and Markov chain Monte Carlo settings
☒	☐	For hierarchical and complex designs, identification of the appropriate level for tests and full reporting of outcomes
☒	☐	Estimates of effect sizes (e.g. Cohen's d, Pearson's r), indicating how they were calculated
☐	☒	Clearly defined error bars<br>*State explicitly what error bars represent (e.g. SD, SE, CI)*

Our web collection on statistics for biologists may be useful.

Software and code

Policy information about availability of computer code

Data collection	No software was used.
Data analysis	Statistical analysis was performed using FlowJo vX. 10.0.8 and GraphPad Prism version 8.0.0 for Windows, GraphPad Software, San Diego, California USA, www.graphpad.com

For manuscripts utilizing custom algorithms or software that are central to the research but not yet described in published literature, software must be made available to editors/reviewers upon request. We strongly encourage code deposition in a community repository (e.g. GitHub). See the Nature Research guidelines for submitting code & software for further information.

Data

Policy information about availability of data

All manuscripts must include a data availability statement. This statement should provide the following information, where applicable:

- Accession codes, unique identifiers, or web links for publicly available datasets
- A list of figures that have associated raw data
- A description of any restrictions on data availability

All data are available within the Article or Supplementary information. Source data are provided with this paper.

Field-specific reporting

Please select the best fit for your research. If you are not sure, read the appropriate sections before making your selection.

☒ Life sciences ☐ Behavioural & social sciences ☐ Ecological, evolutionary & environmental sciences

For a reference copy of the document with all sections, see nature.com/authors/policies/ReportingSummary-flat.pdf

Life sciences study design

All studies must disclose on these points even when the disclosure is negative.

Sample size	Sample sizes were determined to allow the statistical significance of differences of 50% or greater, and according to similar studies conducted in the field. The specific sample size required depended on the experiment. In vitro refer to Nat. Commun. 2019, 10, 3452; Nat. Commun. 2019, 10, 5783; Chem. Mater. 2019, 31, 7212; Sci. Adv. 2020; 6, eaba3546. In vivo refer to Adv. Mater. 2019, 1808278; Adv. Funct. Mater. 2019, 1906623; Adv. Funct. Mater. 2020, 1910176; Nat. Commun. 2018, 9, 1680; Adv. Funct. Mater. 2020, 1909806; Angew. Chem. Int. Ed. 2020, 59, 2.
Data exclusions	No data was excluded.
Replication	All experiments were conducted at least two times and could be reliably reproduced.
Randomization	The samples were divided into different groups randomly in all experiments.
Blinding	Formal blinding was used for ELISA and the TUNEL and H&E staining of tumor tissues.

Reporting for specific materials, systems and methods

Materials & experimental systems

n/a	Involved in the study
☒	☐ Unique biological materials
☐	☒ Antibodies
☐	☒ Eukaryotic cell lines
☒	☐ Palaeontology
☐	☒ Animals and other organisms
☒	☐ Human research participants

Methods

n/a	Involved in the study
☒	☐ ChIP-seq
☐	☒ Flow cytometry
☒	☐ MRI-based neuroimaging

Antibodies

Antibodies used	PerCP-Cyanine5.5-labeled anti-CD3 (BioLegend, catalog: 100328, clone numbers:145-2C11), APC-labeled anti-CD4 (BioLegend, catalog: 100412, clone numbers: GK1.5), PE-Cy7-labeled anti-IFN-γ (BioLegend, catalog: 505826, clone numbers: XMG1.2), and FITC-labeled anti-Ki67 (eBioscience, catalog: 11-5698-82, clone numbers: SolA15).
Validation	PerCP-Cyanine5.5-labeled anti-CD3 Reactivity: Mouse; Host Species: Armenian Hamster; Application: FC - Quality tested. APC-labeled anti-CD4: Reactivity: Mouse; Host Species: Rat; Application: FC - Quality tested. PE-Cy7-labeled anti-IFN-γ: Reactivity: Mouse; Host Species: Rat; Application: FC - Quality tested. FITC-labeled anti-Ki67: Reactivity: Dog, Cynomolgus Monkey, Human, Mouse, Non-human primate, Rat; Rat: Armenian Hamster; Application: WB, IHC IHC(P), ICC/IF, Flow, FN.

Eukaryotic cell lines

Policy information about cell lines

Cell line source(s)	4T1 and 293T were obtained from American type culture collections (ATCC CRL-2539; CRL-3216). H22 was purchased from BeNa Culture Collection (BNCC) (catalog: BNCC338327).
Authentication	Cell lines were not authenticated.
Mycoplasma contamination	No mycoplasma contamination.

Commonly misidentified lines (See ICLAC register)	No commonly misidentified cell lines were used in the study.

Animals and other organisms

Policy information about studies involving animals; ARRIVE guidelines recommended for reporting animal research

Laboratory animals	6-8 weeks old BALB/c female mice (from Jiesijie Laboratory Animal Center) lived under SPF conditions with ambient temperature (~25 °C), humidity (~55%) and 12/12h dark/light cycle.
Wild animals	No wild animals were used in the study.
Field-collected samples	No field collected samples were used in the study.

Flow Cytometry

Plots

Confirm that:

☒ The axis labels state the marker and fluorochrome used (e.g. CD4-FITC).

☒ The axis scales are clearly visible. Include numbers along axes only for bottom left plot of group (a 'group' is an analysis of identical markers).

☒ All plots are contour plots with outliers or pseudocolor plots.

☒ A numerical value for number of cells or percentage (with statistics) is provided.

Methodology

Sample preparation	Tumor tissues were harvested, treated with 1 mg/mL collagenase I (Gibco) for 1 h and ground using the rubber end of a syringe. Cells were filtered through nylon mesh filters and washed with PBS. Cells were further stained with corresponding fluorochrome-conjugated antibodies
Instrument	Beckman CytoFlex
Software	cytExpert
Cell population abundance	At least 10,000 relevant events were acquired for all FACS analysis.
Gating strategy	In general, cells were first gated on FSC/SSC. Singlet cells were gated using FSC-H and FSC-A. Dead cells were then excluded and further surface and intracellular antigen gating was performed on the live cell population.

☒ Tick this box to confirm that a figure exemplifying the gating strategy is provided in the Supplementary Information.
